# Prognostic and Clinicopathological Significance of X-Box-Binding Protein 1 and N-Acetyltransferase 1 in Gallbladder Cancer

**DOI:** 10.3389/fonc.2020.01124

**Published:** 2020-07-24

**Authors:** Rushi Liu, Zhengchun Wu, Yuanfang Zhang, Xiongying Miao, Qiong Zou, Yuan Yuan, Daiqiang Li, Zhulin Yang

**Affiliations:** ^1^Department of Medical Laboratory, Immunodiagnostic Reagents Engineering Research Center of Hunan Province, School of Medicine, Hunan Normal University, Changsha, China; ^2^Hunan Provincial Key Laboratory of Hepatobiliary Disease Research, Department of General Surgery, Second Xiangya Hospital, Central South University, Changsha, China; ^3^Department of Pathology, Third Xiangya Hospital, Central South University, Changsha, China; ^4^Department of Pathology, Second Xiangya Hospital, Central South University, Changsha, China

**Keywords:** gallbladder cancer, X-box-binding protein 1, N-acetyltransferase 1, prognosis, clinicopathological significance

## Abstract

**Background:** X-box-binding protein 1 (XBP1) and N-acetyltransferase 1 (NAT1) are involved in oncogenesis and progression of many human cancer types. However, the roles of XBP1 and NAT1 in gallbladder cancer (GBC) are never reported.

**Methods:** We examined XBP1 and NAT1 expression in GBC and matched adjacent non-tumor tissues via Western blotting. Then, we assayed XBP1 and NAT1 expression in 215 GBCs, including 69 squamous cell/adenosquamous carcinomas (SC/ASCs) and 146 adenocarcinomas (ACs) with immunohistochemistry. Their prognostic and clinicopathological significance was further evaluated using the χ^2^ test or Fisher's exact test, Kaplan–Meier univariate survival analysis, and log-rank tests.

**Results:** XBP1 expression was upregulated, and NAT1 expression was downregulated in GBC. Immunohistochemical results showed that XBP1 expression was negatively associated with NAT1 expression in GBC, including SC/ASC and AC. The rate of patients with an age of more than 45 years, positivity of lymph node metastasis, and invasion were significantly higher in SC/ASC than those in AC (all *P* < 0.05). The percentage of XBP1-positive and NAT1-negative expression was significantly higher in the cases with poor differentiation, advanced tumor, nodes, and metastases (TNM) stage, lymph node metastasis, invasion, and only receiving biopsy in GBC, SC/ASC, and AC (all *P* < 0.05). XBP1-positive and NAT1-negative expression was positively related to larger tumor size (>3 cm) in GBC and AC. There was a negative association between XBP1 and NAT1 expression in GBC, SC/ASC, and AC (all *P* < 0.05). Positive XBP1 and negative NAT1 expression was closely associated with decreased overall survival in GBC, SC/ASC, and AC patients (all *P* < 0.05). The multivariate Cox regression analysis showed that positive XBP1 or negative NAT1 expression was an independent factor for poor prognosis in gallbladder SC/ASC and AC patients.

**Conclusions:** This study indicates that positive XBP1 and negative NAT1 expression are closely associated with the clinicopathological and biological behaviors and poor prognosis in GBC.

## Introduction

Gallbladder cancer (GBC), the most common biliary malignant tumor, is an extremely fatal disease with poor prognosis. The occurrence of GBC exists with geographical and ethnic differences worldwide (1, 2). Histologically, GBC is mainly composed of adenocarcinomas (ACs) and squamous cell/adenosquamous carcinoma (SC/ASC), which account for ~90% and 1–12% of GBC, respectively (3, 4). Due to commonly presenting asymptomatic in early stage, GBC patients are often difficult to be diagnosed at this stage (5, 6). When symptoms such as jaundice and pain occur, patients are mostly in the late stage, with invasion and metastasis, and miss the opportunity to receive radical surgery (6). Nowadays, radical resection remains the only strategy to potentially cure GBC, although there exist adjuvant treatments such as chemotherapy and radiotherapy. Additionally, postoperative adjuvant therapies cannot significantly improve the prognosis of GBC patients (7). Currently, no biomarker can precisely diagnose GBC and predict patients' prognosis, although various studies have been performed (8, 9). Therefore, it is very important to discover reliable markers for early diagnosis and targeted therapy of GBC.

Human X-box-binding protein 1 (XBP1) is a member of the basic region/leucine zipper protein family, which functions as a transcriptional factor and has a zinc finger structure (10). Commonly, XBP1 exists in two isoforms: un-spliced XBP1 and spliced XBP1 (XBP1s). The splicing of un-spliced XBP1 results in the generation of XBP1s, which is catalyzed by inositol-requiring enzyme 1α (IRE1α) (11). XBP1 is involved in maintaining endoplasmic reticulum (ER) homeostasis. When ER stress occurs, the unfolded protein response (UPR) is initiated to recover homeostasis. IRE1α-XBP1 signaling is one of the three major UPR-associated signaling pathways, and IRE1α can mediate the UPR through regulating the expression of XBP1s (12). As a crucial UPR effector, active XBP1s can regulate the expression of various UPR target genes to respond to ER stress (13). XBP1 is related to some human diseases, including cancer. For example, XBP1 is involved in the development and progression of cancer by modulating cancer cell proliferation, apoptosis, invasion, metastasis, and drug resistance (11, 14, 15). Previous studies reported that XBP1 was overexpressed in various human cancer types, such as breast cancer, oral cancer, lung cancer, colorectal cancer, and hepatocellular cancer (14, 16–21). Furthermore, XBP1 overexpression was associated with poor clinicopathological features and clinical outcomes of these cancers. Therefore, XBP1 may be a predictive biomarker of cancer development and progression and a potential therapeutic target. However, the role of XBP1 in GBC is never studied.

Arylamine N-acetyltransferases (NATs) belong to a family of highly conserved enzymes and exist in a number of species, including prokaryotes and eukaryotes (22). In humans, the NAT enzymes are encoded by genes localized on chromosome 8p22 and consist of two members: NAT1 and NAT2 (23). NATs with a size of ~33 kDa are predominantly located in the cytoplasm and composed of 289 amino acids (24). Both NAT1 and NAT2 are involved in acetylation of a series of arylamine, heterocyclic amine, and hydrazine substrates, such as various common carcinogens and therapeutic drugs (25). Thus, NATs are related to carcinogenesis of human cancer, and their genetic polymorphisms are associated with cancer risk (23). As a member of NATs, NAT1 is a cytosolic phase II xenobiotic-metabolizing isozyme that functions as a catalyzer in the biotransformation of various carcinogens and drugs with aromatic and heterocyclic amines (23, 24). More and more evidence indicates that NAT1 is closely associated with the development and progression of human cancers (23, 24, 26, 27). Previous investigations have demonstrated that NAT1 has an effect on biological features of human cancer, such as cell proliferation, invasion, metastasis, and drug resistance and is a biomarker of cancer prognosis (24, 26, 28–30). Nevertheless, NAT1 expression in GBC is never investigated.

Thus, we detected XBP1 and NAT1 expression in GBC in this study. Then, we further evaluated the prognostic and clinicopathological significance of XBP1 and NAT1 in GBC, including SC/ASC and AC.

## Materials and Methods

### Case Selection

We collected 215 GBC tissues, including 69 SC/ASCs and 146 ACs, from January 2001 to December 2013. These tumor tissues were routinely paraffin-embedded. The 69 SC/ASC samples were obtained from Xiangya Hospital, Second Xiangya Hospital, Third Xiangya Hospital, Hunan Provincial People Hospital, Hunan Provincial Tumor Hospital, Changde Central Hospital, and Loudi Central Hospital. The 146 AC samples were obtained from Second Xiangya Hospital and Third Xiangya Hospital. The patients enrolled in this study never received chemotherapy or radiotherapy preoperatively and postoperatively. The subtypes of GBC were diagnosed according to the recommendations of the 7th American Joint Committee on Cancer. Survival information of all patients was obtained through letters and phone calls. The follow-up time was 2 years in this study. The patients surviving more than 2 years were considered as censored cases. This retrospective study was approved by the Ethics Committee for Human Research, Central South University, and was conducted according to the approved guidelines.

### EnVision Immunohistochemistry

The rabbit anti-human XBP1 and NAT1 antibodies were purchased from Santa Cruz Biotechnology (CA, USA). EnVision™ Detection Kit was purchased from Dako Laboratories (CA, USA). The staining of XBP1 and NAT1 was carried out according to the manufacture's protocol. Briefly, the paraffin-embedded tumor tissues were sectioned at 4-μM thick and deparaffinized. Next, the sections were incubated with peroxidase inhibitor (3% H_2_O_2_) for 15 min and treated with sodium citrate buffer for 20 min at 98°C, followed by incubation with primary antibody for 120 min. Then, sections were incubated with solution A (containing horseradish peroxidase-conjugated secondary antibody) for 30 min, followed by 3,3′-diaminobenzidine staining and hematoxylin counter-staining. After being dehydrated with 70–100% alcohol, sections were soaked in xylene for 3 × 5 min, followed by mounting with neutral balsam.

Ten random fields per section were viewed, and the percent of positively stained cells relative to the total number of cells in each section was determined, and the average percentage per case was calculated. At the same time, the strength of staining was rated on a scale of 1–3 (1: no positive staining or uncertainly weak staining; 2: weak to moderate staining; 3: moderate to strong staining). A case was determined as positive XBP1 or NAT1 expression when the average percentage of positively stained cells was ≥10% and staining strength ≥2. The few cases whose percentage of positive staining was 5–10% and staining strength was three were also regarded as positive.

### Western Blot

Total protein was extracted from frozen tissues. Protein concentrations were tested via a bicinchoninic acid protein assay. Protein samples were separated on 10% sodium dodecyl (lauryl) sulfate-polyacrylamide gel electrophoresis gel. The separated proteins were transferred to Immun-Blot polyvinylidene fluoride membrane (Bio-Rad) using a wet transfer system (Bio-Rad) and then incubated with primary antibody at 4°C overnight, followed by incubation with horseradish peroxidase-linked anti-rabbit immunoglobulin G (Merck Millipore) in a dilution of 1:10,000 for 1 h at room temperature. Three primary antibodies were applied in the experiment: XBP1 (1:2,000 dilution; Proteintech, China), NAT1 (1:1,000 dilution; Proteintech, China), and β-actin (1:2,000 dilution; Proteintech, China). Relative protein expression levels were calculated by determining a ratio between the amount of target protein and β-actin.

### Statistical Analysis

Data were analyzed using the statistical package for the Social Sciences Version 23.0 (SPSS 23.0). The inter-relationship of XBP1 or NAT1 expression with histology or clinical factors was determined using χ^2^ or Fisher's exact test. Univariate survival analysis was performed by Kaplan–Meier and time-series test. Multivariate analysis was conducted with a Cox proportional hazards model. A probability level of *P* < 0.05 was considered statistically significant.

## Results

### Characteristics of Patients

The 69 SC/ASC patients comprise 44 females and 25 males (F/M = 1.76), and their ages ranged from 35 to 80 (53.8 ± 10.2) years. The 146 AC patients were composed of 85 females and 61 males (F/M = 1.39), and their age varied from 33 to 78 (52.4 ± 9.6) years old. Histology, the 69 SC/ASCs included 19 (27.5%) well-differentiated tumors, 33 (47.8%) moderately differentiated tumors, and 17 (24.6%) poorly differentiated tumors. The 146 ACs consisted of 51 (34.9%) well-differentiated tumors, 54 (37.0%) moderately differentiated tumors, and 41 (28.1%) poorly differentiated tumors. Among the 69 SC/ASC patients, 42 (60.9%) and 45 (65.2%) presented regional lymph node metastasis and invasion to surrounding tissues and organs, respectively. Among the 146 AC patients, 66 (45.2%) and 74 (50.7%) presented lymph node metastasis and invasion, respectively. Gallstone was observed in 38 (55.1%) SC/ASC patients and 68 (46.6%) AC patients. Among the 215 GBC patients, 29 SC/ASC patients and 77 AC patients were in a TNM stage of I + II, and 40 SC/ASC patients and 69 AC patients were in a TNM stage of III + IV. Among the 69 SC/ASC patients, 27 patients received radical resection, 28 patients received palliative surgery, and 14 patients only received biopsy (Table 1). Among 146 AC patients, 75 patients received radical resection, 50 patients received palliative surgery, and 21 patients only received biopsy (Table 1).

**Table 1:** Comparison of gallbladder SC/ASC and AC clinicopathological features, including XBP1 and NAT1 expression.

**Clinicopathological characteristics**	**Number of SC/ASC (%)**	**Number of AC (%)**	**χ^2^**	***P***
**Gender**				
Male	25 (36.2)	61 (41.8)	0.601	0.438
Female	44 (63.8)	85 (58.2)		
**Age**				
≤45 years	3 (4.3)	20 (13.7)	4.289	0.038
>45 years	66 (95.7)	126 (86.3)		
**Differentiation**				
Well	19 (27.5)	51 (34.9)	2.235	0.308
Moderate	33 (47.8)	54 (37.0)		
Poor	17 (24.6)	41 (28.1)		
**Maximum Tumor Diameter**				
≤3 cm	39 (56.5)	90 (61.6)	0.512	0.474
>3 cm	30 (43.5)	56 (38.4)		
**Cholecystolithiasis**				
No	31 (44.9)	78 (53.4)	1.353	0.245
Yes	38 (55.1)	68 (46.6)		
**TNM Stages**				
I + II	29 (42.0)	77 (52.7)	2.151	0.143
III + IV	40 (58.0)	69 (47.3)		
**Lymph Node Metastasis**				
No	27 (39.1)	80 (54.8)	4.599	0.032
Yes	42 (60.9)	66 (45.2)		
**Locoregional Invasion**				
No	24 (34.8)	72 (49.3)	4.004	0.045
Yes	45 (65.2)	74 (50.7)		
**Surgical Methods**				
Radical	27(39.1)	75 (51.4)	3.002	0.223
Palliative	28 (40.6)	50 (34.2)		
Biopsy	14 (20.3)	21 (14.4)		
**XBP1**				
–	25 (36.2)	67 (45.9)	0.985	0.337
+	44 (63.8)	79 (54.1)		
**NAT1**				
–	42 (60.9)	80 (54.8)	0.706	0.443
+	27 (39.1)	66 (45.2)		

*–, negative expression; +, positive expression*.

### X-Box-Binding Protein 1 Was Overexpressed, and N-Acetyltransferase 1 Was Down-Expressed in Gallbladder Cancer Tissue

**Figure 1:**
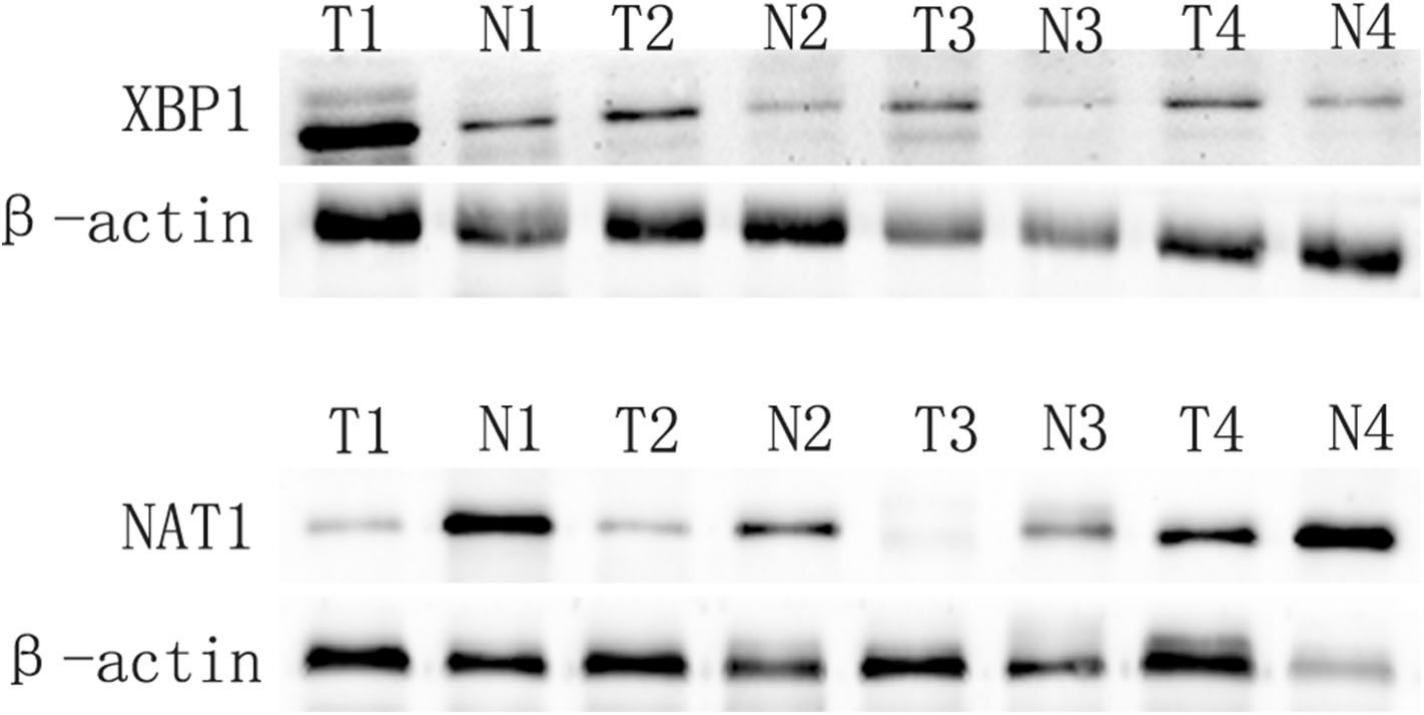
XBP1 expression is upregulated, and NAT1 expression is downregulated in GBC. *T* and *N* represented cancer tissues and corresponding adjacent non-tumor tissues, respectively.

We applied Western blot to examine the expression of XBP1 and NAT1 in GBC and matched adjacent non-tumor tissues. Our data showed that XBP1 was overexpressed in GBC tissues compared with matched adjacent non-tumor tissues. Conversely, NAT1 was down-expressed in GBC tissues in comparison with corresponding non-tumor tissues (Figure 1).

### Comparison of Clinicopathological Characteristics in Gallbladder Squamous Cell/Adenosquamous Carcinomas and Adenocarcinoma

In this study, we found that the rate of SC/ASC patients with lymph node metastasis and invasion to surrounding tissues and organs was significantly higher compared with that of the AC patients (*P* = 0.032, *P* = 0.045). SC/ASC was more likely to occur in patients with the age of more than 45 years compared with AC (*P* = 0.038). Between SC/ASC and AC, there was no significant difference in other clinicopathological features, including gender, tumor differentiation, tumor size, the occurrence of gallstone, TNM staging, receiving surgical methods, and XBP1 and NAT1 expression (*P* > 0.05, as shown in Table 1).

### Immunohistochemistry Reveals That X-Box-Binding Protein 1 Expression Is Negatively Correlated to X-Box-Binding Protein 1 Expression in Gallbladder Cancer

**Figure 2:**
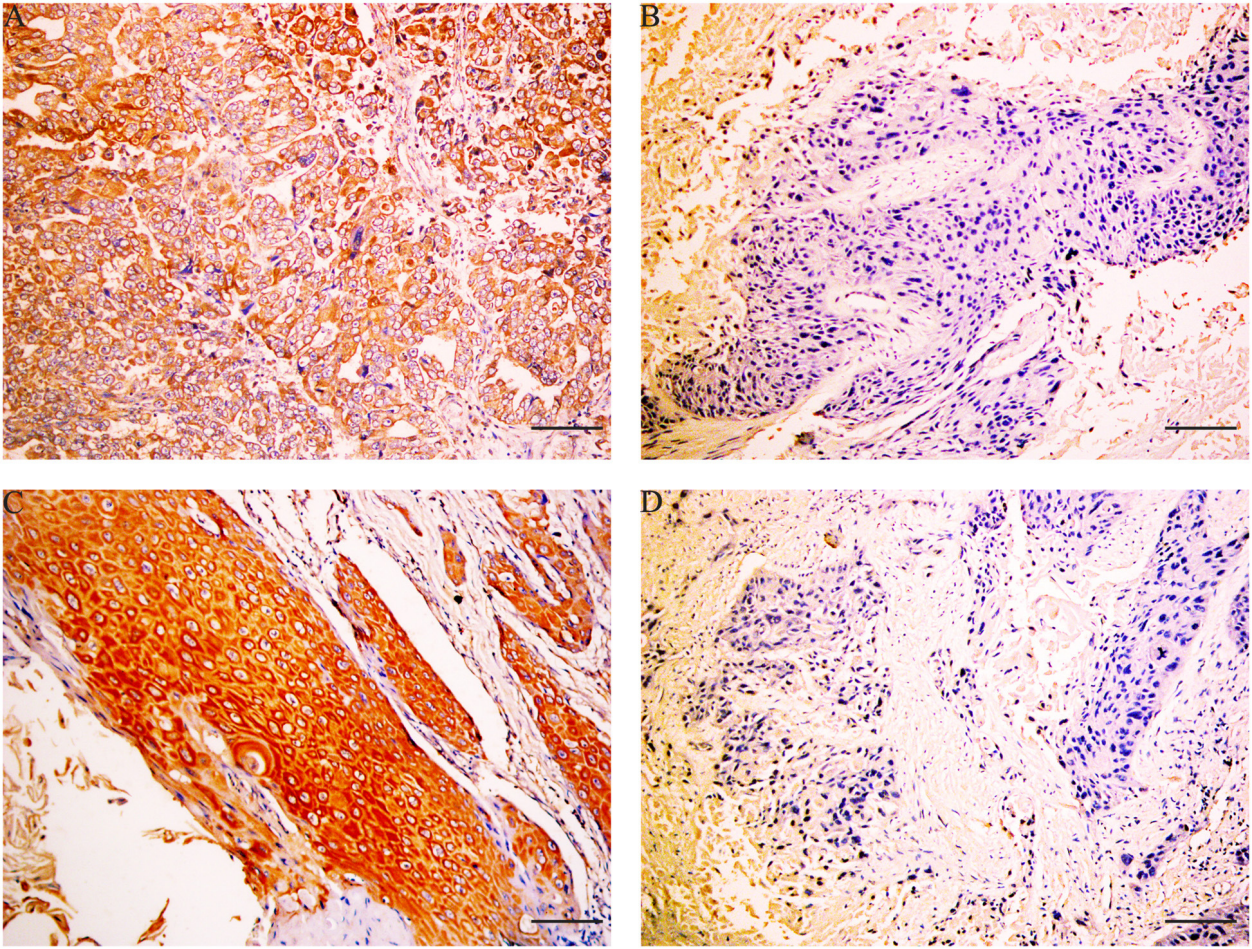
XBP1 and NAT1 expression in gallbladder ASC/SC, original magnification ×200. XBP1 expression was localized in the nuclei, and NAT1 expression was localized in the cytoplasm and/or nuclei. **(A)** Positive expression of XBP1 in poorly differentiated SC. **(B)** Negative expression of XBP1 in moderately differentiated SC. **(C)** Positive expression of NAT1 in well-differentiated ASC. **(D)** Negative expression of NAT1 in moderately differentiated ASC. Scale bars: 50 μm.

**Figure 3:**
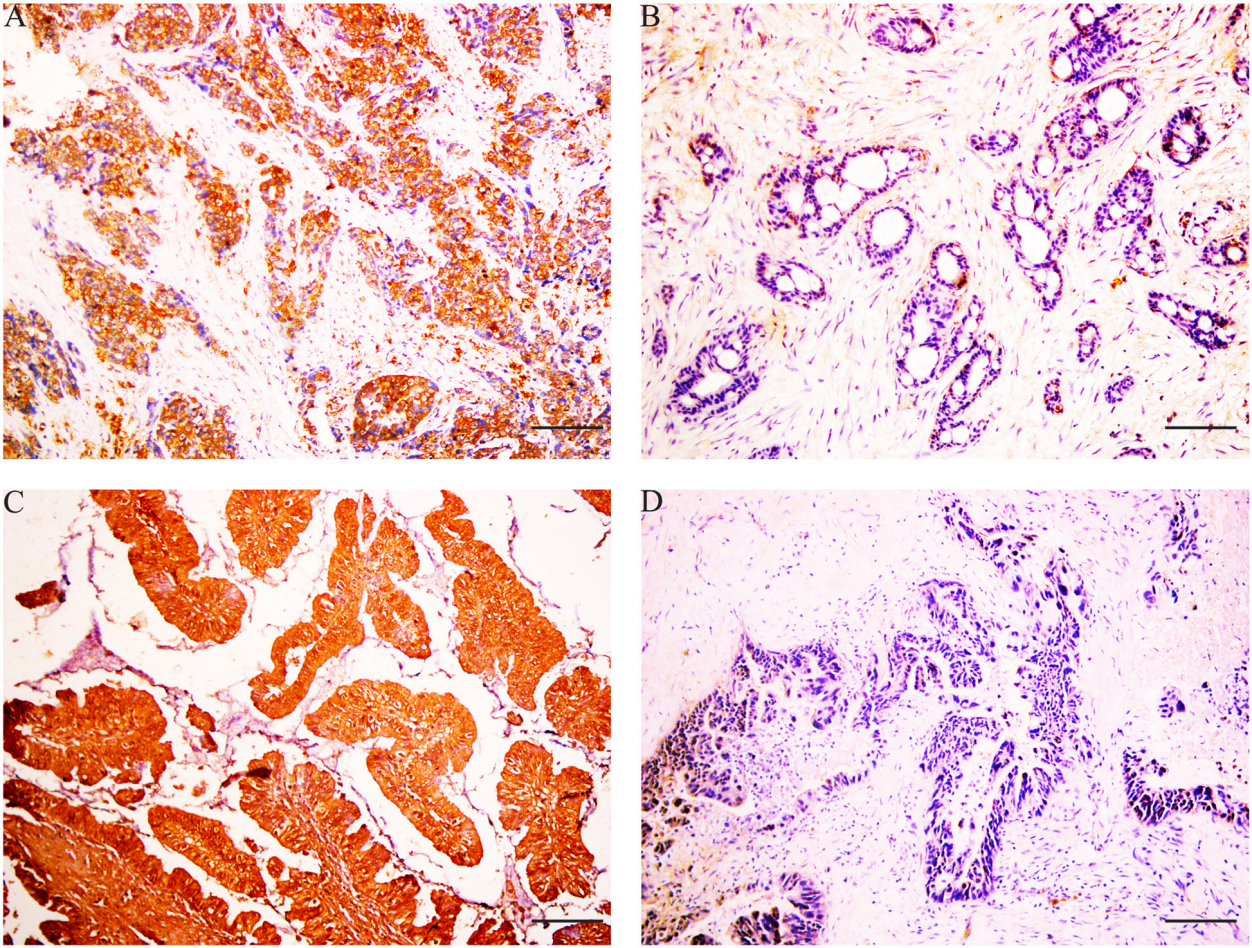
XBP1 and NAT1 expression in gallbladder AC, original magnification ×200. XBP1-positive reactions were localized in the nuclei, and NAT1-positive reactions were localized in the cytoplasm. **(A)** Positive expression of XBP1 in poorly differentiated AC. **(B)** Negative expression of XBP1 in well-differentiated AC. **(C)** Positive expression of NAT1 in well-differentiated AC. **(D)** Negative expression of NAT1 in moderately differentiated AC. Scale bars: 50 μm.

Immunohistochemical results showed that the majority of XBP1-positive reactions were localized in the nuclei of SC/ASC and AC, and NAT1-positive reactions were localized in the cytoplasm of the SC/ASC (Figure 2) and AC (Figure 3). Furthermore, the association of XBP1 and NAT1 expression was evaluated in GBC. In GBC, 37 cases presented NAT1-positive expression among the 123 cases with XBP1-positive expression, whereas 36 cases showed NAT1-negative expression among the 92 XBP1-negative cases (Table 2, *P* < 0.01). In SC/ASC, 12 cases exhibited NAT1-positive expression among the 44 cases with XBP1-positive expression, whereas 10 cases showed NAT1-negative expression among the 25 cases with XBP1-negative expression (Table 3, *P* < 0.01). In AC, NAT1 was positively expressed in 25 cases among the 79 cases with XBP1-positive expression, whereas NAT1 was negatively expressed in 26 cases among the 67 AC cases with XBP1-negative expression (Table 4, *P* < 0.01). These results suggested that these two marker expressions were negatively correlated in GBC, SC/ASC, and AC.

**Table 2:** Association between XBP1 expression and NAT1 expression in GBC.

	**XBP1**	**NAT1**		**Total**
		**–**	**+**	
	–	36	56	92
	+	86	37	123
Total		122	93	215

*χ^2^ = 20.326, P = 0.000; –, negative expression; +, positive expression*.

**Table 3:** Association between XBP1 expression and NAT1 expression in SC/ASC.

	**XBP1**	**NAT1**		**Total**
		**–**	**+**	
	–	10	15	25
	+	32	12	44
Total		42	27	69

*χ^2^ = 7.169, P = 0.007; –, negative expression; +, positive expression*.

**Table 4:** Association between XBP1 expression and NAT1 expression in AC.

	**XBP1**	**NAT1**		**Total**
		**–**	**+**	
	–	26	41	67
	+	54	25	79
Total		80	66	146

*χ^2^ = 12.779, P = 0.000; –, negative expression; +, positive expression*.

### Association of Clinicopathological Characteristics and X-Box-Binding Protein 1/N-Acetyltransferase 1 Expression in Gallbladder Cancer Patients

We evaluated the clinicopathological significance of XBP1 and NAT1 expression in CBC. Both positive XBP1 expression and negative NAT1 expression were significantly associated with the poorly differentiated type, larger tumor size (>3 cm), advanced TNM stage (III + IV), lymph node metastasis, invasion, and only receiving biopsy in GBC (all *P* < 0.05; Tables 5, 6). The expression of XBP1 and NAT1 was non-significantly related to the occurrence of gallstone in GBC (all *P* > 0.05).

**Table 5:** Correlations of XBP1 expression with the clinicopathological characteristics of GBC, SC/ASC, and AC.

**Clinicopathological characteristics**	**GBC**			**SC/ASC**			**AC**		
	**Number of patients**	**Pos No. (%)**	***P***	**Number of patients**	**Pos No. (%)**	***P***	**Number of patients**	**Pos No. (%)**	***P***
**Differentiation**									
Well	70	29 (41.4)	0.000	19	8 (42.1)	0.033	51	21 (41.2)	0.008
Moderately	87	50 (57.5)		33	22 (66.7)		54	28 (51.9)	
Poorly	58	44 (75.9)		17	14 (82.4)		41	30 (73.2)	
**Tumor size**									
≤3 cm	129	63 (48.8)	0.002	39	24 (61.5)	0.742	90	39 (43.3)	0.001
>3 cm	86	60 (69.8)		30	20 (66.7)		56	40 (71.4)	
**Gallstone**									
No	109	66 (60.6)	0.315	31	23 (74.2)	0.104	78	43 (55.1)	0.791
Yes	106	57 (53.8)		38	21 (55.3)		68	36 (52.9)	
**Lymph node metastasis**									
No	107	43 (40.2)	0.000	27	11 (40.7)	0.001	80	32 (40.0)	0.000
Yes	108	80 (74.1)		42	33 (78.6)		66	47 (71.2)	
**Invasion**									
No	96	33 (34.4)	0.000	24	8 (33.3)	0.000	72	25 (34.7)	0.000
Yes	119	90 (75.6)		45	36 (80.0)		74	54 (72.9)	
**TNM stage**									
I + II	106	26 (24.5)	0.000	29	11 (37.9)	0.000	77	26 (33.8)	0.000
III + IV	109	88 (80.7)		40	33 (82.5)		69	53 (76.8)	
**Surgery**									
Radical	102	39 (38.2)	0.000	27	12 (44.4)	0.018	75	27 (36.0)	0.000
Palliative	78	54 (69.2)		28	20 (71.4)		50	34 (68.0)	
Biopsy	35	30 (85.7)		14	12 (85.7)		21	18 (85.7)	

**Table 6:** Correlations of NAT1 protein expression with the clinicopathological characteristics of GBC, SC/ASC, and AC.

**Clinicopathological characteristics**	**GBC**			**SC/ASC**			**AC**		
	**Number of patients**	**Pos No. (%)**	***P***	**Number of patients**	**Pos No. (%)**	***P***	**Number of patients**	**Pos No. (%)**	***P***
**Differentiation**									
Well	70	42 (60.0)	0.001	19	12 (63.2)	0.018	51	30 (58.8)	0.043
Moderately	87	34 (39.1)		33	12 (36.4)		54	22 (40.7)	
Poorly	58	17 (29.3)		17	3 (17.6)		41	14 (34.1)	
**Tumor size**									
≤3 cm	129	64 (49.6)	0.021	39	14 (35.9)	0.643	90	50 (55.6)	0.001
>3 cm	86	29 (33.7)		30	13 (43.3)		56	16 (28.6)	
**Gallstone**									
No	109	42 (38.5)	0.156	31	8 (25.8)	0.041	78	34 (43.6)	0.674
Yes	106	51 (48.1)		38	19 (50.0)		68	32 (47.1)	
**Lymph node metastasis**									
Negative	107	68 (63.6)	0.000	27	20 (74.1)	0.000	80	48 (60.0)	0.000
Positive	108	25 (23.1)		42	7 (16.7)		66	18 (27.3)	
**Invasion**									
Negative	96	47 (49.0)	0.000	24	1 (75.0)	0.000	72	46 (63.9)	0.000
Positive	119	29 (24.4)		45	9 (20.0)		74	20 (27.0)	
**TNM stage**									
I + II	106	67 (63.2)	0.000	29	19 (65.5)	0.000	77	48 (62.3)	0.000
III + IV	109	26 (23.9)		40	8 (20.0)		69	18 (26.1)	
**Surgery**									
Radical	102	61 (59.8)	0.000	27	16 (59.2)	0.005	75	45 (60.0)	0.000
Palliative	78	28 (35.9)		28	10 (35.7)		50	18 (36.0)	
Biopsy	35	4 (11.4)		14	1 (7.1)		21	3 (14.3)	

*Pos No., positive number*.

Furthermore, we analyzed the clinicopathological significance of XBP1 and NAT1 expression in SC/ASC and AC. Both XBP1-positive expression and NAT1-negative expression were significantly correlated to poorly differentiated types, advanced TNM stages, lymph node metastasis, invasion, and only receiving biopsy in SC/ASC (all *P* < 0.05; Tables 5, 6). The rate of NAT1-positive expression was significantly higher in patients with gallstone compared with that in patients without gallstone (*P* = 0.041). The expression of XBP1 was non-significantly related to the occurrence of gallstone in SC/ASC (*P* = 0.104).

Similarly, XBP1-positive expression and NAT1-negative expression were closely related to poorly differentiated type, larger tumor mass, advanced TNM stage (III + IV), lymph node metastasis, invasion, and only receiving biopsy in AC (all *P* < 0.05; Tables 5, 6). The expression of XBP1 and NAT1 was non-significantly associated with the occurrence of gallstone in AC (all *P* > 0.05).

These results suggested that the clinicopathological significance of XBP1 expression was opposite to NAT1 expression in GBC, SC/ASC, and AC.

### Correlation Between Survival Rates and X-Box-Binding Protein 1/N-Acetyltransferase 1 Expression in Patients With Gallbladder Cancer

Survival analysis of all patients was performed. Among the 146 AC patients, there were 57 surviving more than 1 year (25 more than 2 years) and 89 surviving <1 year. Among the 69 SC/ASC patients, 16 survived more than 1 year (6 more than 2 years), and 53 survived <1 year.

**Figure 4:**
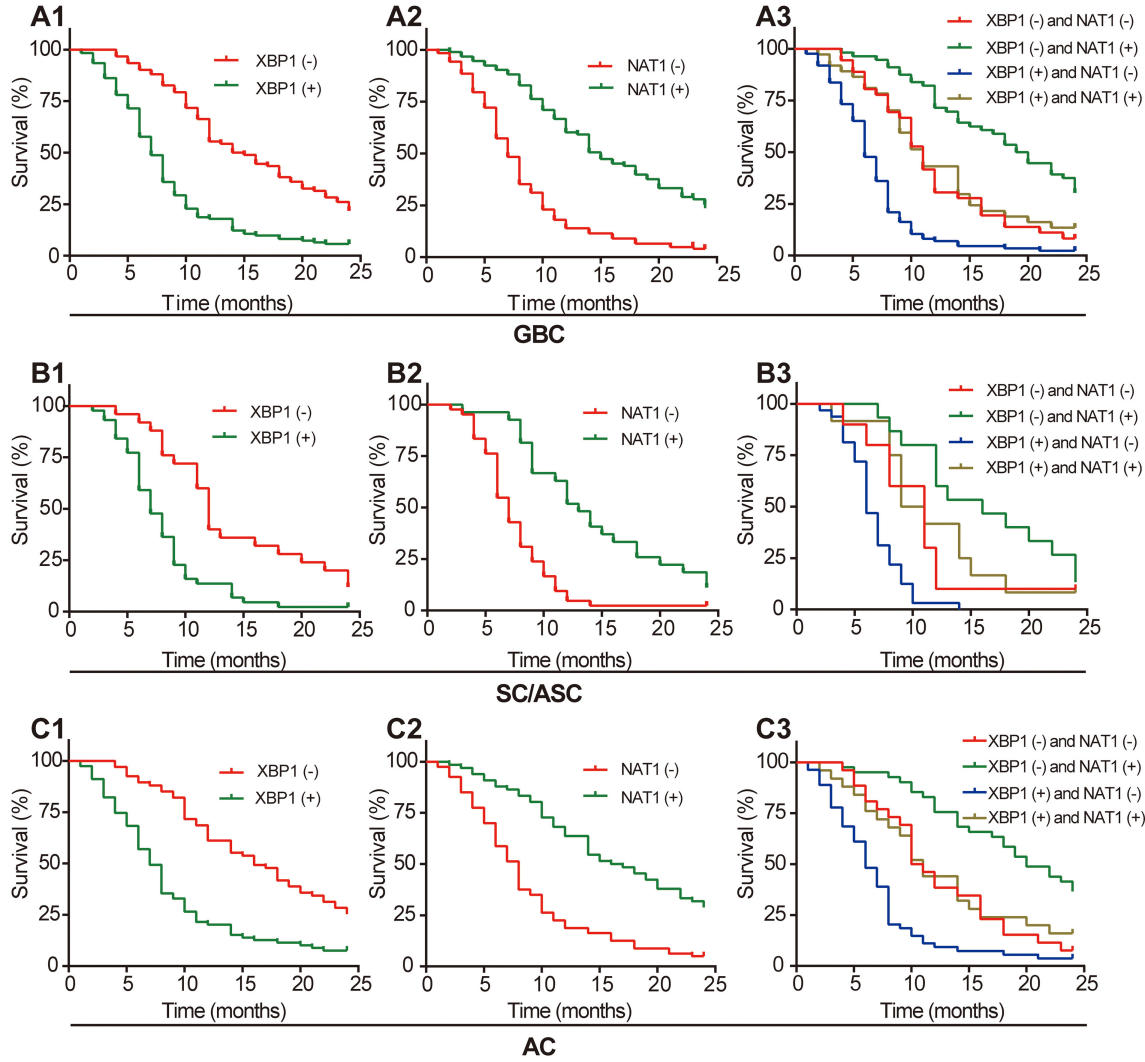
Positive XBP1 expression and negative NAT1 expression are associated with poor prognosis of GBC, SC/ASC, and AC patients. **(A1)** Kaplan–Meier plots of overall survival in GBC patients with positive or negative XBP1 expression. **(A2)** Kaplan–Meier plots of overall survival in GBC patients with positive or negative NAT1 expression. **(A3)** Kaplan–Meier plots of overall survival in GBC patients with XBP1 and NAT1 expression. **(B1)** Kaplan–Meier plots of overall survival in SC/ASC patients with positive or negative XBP1 expression. **(B2)** Kaplan–Meier plots of overall survival in SC/ASC patients with positive or negative NAT1 expression. **(B3)** Kaplan–Meier plots of overall survival in SC/ASC patients with XBP1 and NAT1 expression. **(C1)** Kaplan–Meier plots of overall survival in AC patients with positive or negative XBP1 expression. **(C2)** Kaplan–Meier plots of overall survival in AC patients with positive or negative NAT1 expression. **(C3)** Kaplan–Meier plots of overall survival in AC patients with XBP1 and NAT1 expression.

As shown in Table 7, the average survival time of GBC patients (including SC/ASC and AC) was significantly related to several clinicopathological parameters, including tumor differentiation, tumor size, TNM stage, lymph node metastasis, invasion, receiving the surgical procedure, and XBP1 and NAT1 expression (all *P* < 0.05). Additionally, the existence of gallstone was closely associated with the average survival time of SC/ASC patients (*P* = 0.008). Kaplan–Meier survival curves showed that the overall survival time of patients with XBP1-negative or NAT1-positive expression was significantly longer than that of patients with XBP1-positive or NAT1-negative expression in GBC, SC/ASC, and AC (Figure 4).

**Table 7:** Association of clinicopathological characteristics (including XBP1 and NAT1 expression) with average survival of GBC, SC/ASC, and AC patients.

**Clinicopathological characteristics**	**GBC**			**SC/ASC**			**AC**		
	**Sample (*n*)**	**AS (month)**	***P***	**Sample (*n*)**	**AS (month)**	***P***	**Sample (*n*)**	**AS (month)**	***P***
**Differentiation**									
Well	70	15.84 (5-24)	0.000	19	13.68 (5-24)	0.000	51	16.69 (5-24)	0.000
Moderately	87	11.63 (2-24)		33	11.58 (4-24)		54	12.33 (2-24)	
Poorly	58	6.38 (1-24)		17	6.12 (2-14)		41	6.49 (1-24)	
**Tumor size**									
≤3 cm	129	12.43 (1-24)	0.026	30	14.57 (6-24)	0.000	90	14.60 (1-24)	0.000
>3 cm	86	10.31 (1-24)		39	7.44 (2-24)		56	8.38 (1-24)	
**Gallstones**									
No	109	11.07 (2-24)	0.371	31	8.26 (3-18)	0.008	78	12.19 (2-24)	0.980
Yes	106	12.11 (1-24)		38	12.90 (2-24)		68	12.24 (1-24)	
**TNM stage**									
I + II	106	16.44 (3-24)	0.000	29	16.31 (3-24)	0.000	77	16.99 (3-24)	0.000
III + IV	109	6.86 (1-14)		40	6.83 (2-14)		69	6.88 (1-24)	
**Lymph node metastasis**									
No	107	15.92 (2-24)	0.000	27	16.04 (3-24)	0.000	80	16.35 (2-24)	0.000
Yes	108	7.30 (1-24)		42	7.45 (2-15)		66	7.20 (1-24)	
**Invasion**									
No	96	17.48 (3-24)	0.000	24	17.25 (3-24)	0.000	72	18.08 (4-24)	0.000
Yes	119	6.83 (1-24)		45	7.38 (2-20)		74	6.50 (1-14)	
**Surgery**									
Radical	102	17.23 (5-24)	0.000	27	16.93 (5-24)	0.000	75	17.84 (6-24)	0.000
Palliative	78	7.03 (1-14)		28	7.32 (2-12)		50	6.86 (1-14)	
Biopsy	35	5.31 (1-9)		14	6.00 (4-8)		21	4.86 (1-9)	
**XBP1**									
–	92	15.58 (4-24)	0.000	25	14.08 (4-24)	0.000	67	16.13 (4-24)	0.000
+	123	8.60 (1-24)		44	8.09 (2-24)		79	8.89 (1-24)	
**NAT1**									
–	122	8.48 (1-24)	0.000	42	7.64 (2-24)	0.001	80	8.93 (1-24)	0.000
+	93	15.66 (2-24)		27	14.33 (3-24)		66	16.20 (2-24)	
**XBP1 and NAT1**									
XBP1(–) and NAT1(–)	36	12.00 (4-24)	0.000	10	10.07 (4-24)	0.000	26	12.50 (4-24)	0.000
XBP1(–) and NAT1(+)	56	17.86 (4-24)		15	16.33 (7-24)		41	18.44 (4-24)	
XBP1(+) and NAT1(–)	86	7.01 (1-24)		32	6.67 (2-14)		54	7.20 (1-24)	
XBP1(+) and NAT1(+)	37	12.30 (2-24)		12	11.83 (3-24)		25	12.52 (2-24)	

*–, negative expression; +, positive expression; AS, average survival*.

Furthermore, we defined four groups based on the expression of XBP1 and NAT1 in GBC, SC/ASC, and AC (XBP1-negative/NAT1-negative expression, XBP1-negative/NAT1-positive expression, XBP1-positive/NAT1-negative expression, and XBP1-positive/NAT1-positive expression). Kaplan–Meier survival curves showed that the group with XBP1-negative and NAT1-positive expression had the longest overall survival time, and the group with XBP1-positive and NAT1-negative expression had the shortest overall survival time in GBC, SC/ASC, and AC (Figure 4).

Cox's multivariate analysis demonstrated that the poor differentiation, tumor size ≥3 cm, advanced TNM stages (III + IV), lymph node metastasis, invasion, and only receiving biopsy were negatively correlated with overall survival of SC/ASC and AC patients, suggesting these parameters are risk factors and independent prognostic predictors of SC/ASC and AC (Table 8). XBP1-positive expression was negatively associated with the overall survival of SC/ASC and AC patients, suggesting that XBP1-positive expression functions as a risk role in SC/ASC and AC (Table 8). On the contrary, NAT1-positive expression was positively related to the overall survival of SC/ASC and AC patients, indicating that NAT1-positive expression plays a protective role in SC/ASC and AC (Table 8). Thus, our results revealed that XBP1-positive expressions and NAT1-negative expression are independent prognostic biomarkers of SC/ASC and AC patients.

**Table 8:** Multivariate Cox regression analysis of survival rate in SC/ASC and AC patients.

**Groups**	**Factors**	**SC/ASC**		**AC**	
		***P***	**HR (95%CI)**	***P***	**HR (95%CI)**
Differentiated degree	Well/moderately/poorly	0.008	1.813 (1.166–2.818)	0.001	1.567 (1.193–2.057)
Tumor size	≤3 cm/>3 cm	0.022	2.560 (1.148–5.707)	0.012	1.865 (1.149–3.026)
Gallstone	No/Yes	0.047	1.770 (1.009–3.107)	0.005	1.728 (1.181–2.528)
TNM stage	I + II/III + IV	0.023	2.504 (1.134–5.528)	0.017	2.399 (1.171–4.915)
Lymph node metastasis	No/Yes	0.018	3.155 (1.222–8.147)	0.011	2.394 (1.220–4.698)
Invasion	No/Yes	0.027	3.394 (1.150–10.013)	0.000	5.795 (2.913–11.530)
Surgery	Radical/Palliative/Biopsy	0.006	2.192 (1.254–3.833)	0.000	2.540 (1.679–3.840)
XBP1	–/+	0.012	2.305 (1.205–4.409)	0.003	1.848 (1.227–2.783)
NAT1	–/+	0.015	0.351 (0.151–0.813)	0.000	0.472 (0.312–0.714)

*–, negative expression; +, positive expression; HR, hazard ratio; CI, confident interval*.

## Discussion

Compared with the ordinary AC, the SC/ASC is a relatively rare subtype of GBC. Because of the low incidence of gallbladder SC/ASC, there is a lack of enough cases included in previous studies to completely investigate the clinicopathological and biological characteristics of SC/ASC. To date, most studies regarding the clinicopathological characteristics of SC/ASC are individual case studies or analyses of small case series. As we know, 69 cases are relatively large samples in current studies of SC/ASC so that more detail and accurate information about SC/ASC can be gained from this study. Clinicopathological characteristics of SC/ASC are often compared with those of AC. Regarding the occurrence of lymph node metastasis in SC/ASC and AC, there exist contrary opinions in previous reports. Kondo et al. (31) reported that the incidence of lymph node metastasis in SC/ASC was significantly lower compared with that in AC, but Kim et al. (32) found a reverse result. Our results showed that lymph node metastasis and invasion easily occur in SC/ASC compared with those in AC. The previously discussed contradictory results may be attributed to the difference in sample size and geographical and ethnic differences. Additionally, we found that other clinicopathological features, including tumor differentiated degree, tumor size, the occurrence of gallstone, TNM stages, and XBP1 and NAT1 expression were non-significantly different in SC/ASC and AC. Therefore, our results suggested that the clinicopathological and biological characteristics of the gallbladder SC/ASC were alike to the gallbladder AC, which is consistent with previous reports.

GBC possesses a highly malignant degree and poor overall survival. In this study, we found that early TNM stages, tumor size <3 cm, no lymph node metastasis, and no invasion were positively correlated with the average survival time of GBC patients. Therefore, early diagnosis is the key to improving the prognosis of GBC. However, early diagnosis is difficult for GBC patients. Although various studies were performed to find diagnostic marks for GBC, GBC remains lacking in specific diagnostic marks. Biological marks XBP1 and NAT1 are associated with progression and prognosis of a variety of human cancer types, but their roles in GBC remain to be identified. Thus, this study investigated the expression of XBP1 and NAT1 and evaluated their clinicopathological and prognostic significance in GBC, including SC/ASC and AC. Previous studies have demonstrated that XBP1 expression is correlated with NAT1 expression (33), and XBP1 and NAT1 may share a common transcriptional network in breast cancer (34). Likewise, our data showed that XBP1 expression was negatively associated with NAT1, and the clinicopathological and prognostic significance of XBP1 expression was inversed to NAT1 expression in GBC. Thus, we speculated that XBP1 and NAT1 might have an association in the development and progression of GBC, which remains to be fully elucidated.

XBP1 functions as a transcription factor and is involved in tumorigenesis. There is increasing evidence demonstrating that XBP1 may be a potential oncogenic gene (11, 35). Through multiply molecular mechanisms, XBP1 plays a role in regulating the biology of cancer cells, such as proliferation, invasion, migration, apoptosis, and drug resistance (11, 15). XBP1 presents overexpressed in various certain human cancer tissues and plays an oncogenic role in carcinogenesis and progression. XBP1 expression is upregulated in breast cancer, oral cancer, colorectal cancer, lung cancer, hepatocellular cancer, osteosarcoma, and esophageal cancer tissues, compared with matched non-tumor tissues (14, 16–21). Likewise, we found that XBP1 was upregulated in GBC tissues compared with adjacent non-tumor tissues. Additionally, XBP1 overexpression is closely associated with clinicopathological features and poor prognosis of multiple human cancers (14, 16–21). For example, Sun et al. (17) reported that high XBP1 expression was significantly related to histological grade III, advanced clinical stages (stages III and IV), and poor prognosis in oral SC carcinoma. Similarly, our data also revealed that positive XBP1 expression was correlated with poor clinicopathological features of GBC, including poorly differentiated type, lymph node metastasis, invasion, and advanced TNM stages (III or IV). Thus, we speculated that XBP1 might participate in tumorigenesis and progression of GBC, which needs further experiments to validate.

NAT1 is a xenobiotic-metabolizing enzyme that is involved in the activation or deactivation of environmental carcinogens (25). Recently, many studies have been concentrated on the role of NAT1 in the tumorigenesis and progression of human cancers. It has been reported that NAT1 is a novel drug target in cancer development (24). NAT1 plays different roles in different human cancer types or different tumor stages. NAT1 expression also varies from human cancer types and tumor stages. For example, in breast cancer, NAT1 expression is downregulated in young patients (≤45 years) compared with that in old patients (36), whereas NTA1 presents overexpressed in estrogen receptor-positive cancer in comparison of estrogen receptor-negative cancer (29). NAT1 exhibits higher expression in brain cancer tissues than that in normal tissue (37), whereas NAT1 expression was significantly less in colorectal cancer tissues than that in normal tissues and adjacent non-cancerous tissues (38, 39). Additionally, the difference of NAT1 expression between prostate cancer and normal tissue varied from tumor grade (40). In this study, we firstly identified that NAT1 was down-expressed in GBC tissues compared with that in adjacent non-tumor tissues, which is similar to previous reports (38–40). This difference in NAT1 expression in cancer may be attributed to organ-specific differences. We further evaluated the clinicopathological significance of NAT1 expression. We found that negative NAT1 expression was positively correlated with several malignant clinicopathological parameters of GBC, including poorly differentiated type, larger tumor size (>3 cm), lymph node metastasis, invasion, advanced TNM stage, which is consistent with other reports (26). Furthermore, previous studies have revealed that NAT1 is a prognostic biomarker of cancer. Minchin et al. (29) found that low NAT1 mRNA expression was significantly associated with poor survival in breast cancer patients and was related to chemotherapy resistance. Similarly, other studies also reported that breast cancer patients with NAT1-negative expression have a poor prognosis compared with those with NAT1-positive expression (26, 41). Consistent with these previous studies, our data showed that patients with NAT1-negative expression had poor prognosis than those with NAT1-positive expression. Thus, this study suggested that NAT1 might play an important role in the development and progression of GBC, which needs further experiments to validate and explore the underlying molecular mechanism.

To our best knowledge, this study firstly reported XBP1 and NAT1 expression and their clinicopathological and prognostic significance in GBC. We firstly found that XBP1 and NAT1 have a negative association in expression and clinicopathological significance in GBC, which is never reported in other human cancers. This study revealed that XBP1-positive expression and NAT1-negative expression were associated with malignant clinicopathological behavior and poor prognosis of GBC. Furthermore, Cox's regression analysis showed that positive XBP1 and negative NAT1 expression were identified as independent factors for poor prognosis in gallbladder SC/ASC and AC patients. Thus, XBP1 and NAT1 may be involved in the carcinogenesis and development of GBC, and more studies are needed to explore the potential molecular mechanisms.

In conclusion, this study indicated that positive XBP1 and negative NAT1 expression were closely associated with the clinicopathological and biological behaviors and poor prognosis in GBC.

## Data Availability Statement

The datasets generated for this study are available on request to the corresponding author.

## Ethics Statement

The studies involving human participants were reviewed and approved by The Ethics Committee for Human Research, Central South University. The patients/participants provided their written informed consent to participate in this study.

## Author Contributions

RL, ZW, and YZ performed the experiments and wrote the paper. ZY designed the study and revised the paper. ZY and XM performed the statistical analysis. DL, YY, and QZ collected specimens and experimental materials. All authors read and approved the final manuscript.

## Conflict of Interest

The authors declare that the research was conducted in the absence of any commercial or financial relationships that could be construed as a potential conflict of interest.
